# A Comparison of Methods for Clustering 16S rRNA Sequences into OTUs

**DOI:** 10.1371/journal.pone.0070837

**Published:** 2013-08-13

**Authors:** Wei Chen, Clarence K. Zhang, Yongmei Cheng, Shaowu Zhang, Hongyu Zhao

**Affiliations:** 1 College of Automation, Northwestern Polytechnical University, Xi'an, Shaanxi, China; 2 Department of Biostatistics, School of Public Health, Yale University, New Haven, Connecticut, United States of America; 3 Keck Biotechnology Laboratory, Biostatistics Resource, School of Medicine, Yale University, New Haven, Connecticut, United States of America; University of Milan-Bicocca, Italy

## Abstract

Recent studies of 16S rRNA sequences through next-generation sequencing have revolutionized our understanding of the microbial community composition and structure. One common approach in using these data to explore the genetic diversity in a microbial community is to cluster the 16S rRNA sequences into Operational Taxonomic Units (OTUs) based on sequence similarities. The inferred OTUs can then be used to estimate species, diversity, composition, and richness. Although a number of methods have been developed and commonly used to cluster the sequences into OTUs, relatively little guidance is available on their relative performance and the choice of key parameters for each method. In this study, we conducted a comprehensive evaluation of ten existing OTU inference methods. We found that the appropriate dissimilarity value for defining distinct OTUs is not only related with a specific method but also related with the sample complexity. For data sets with low complexity, all the algorithms need a higher dissimilarity threshold to define OTUs. Some methods, such as, CROP and SLP, are more robust to the specific choice of the threshold than other methods, especially for shorter reads. For high-complexity data sets, hierarchical cluster methods need a more strict dissimilarity threshold to define OTUs because the commonly used dissimilarity threshold of 3% often leads to an under-estimation of the number of OTUs. In general, hierarchical clustering methods perform better at lower dissimilarity thresholds. Our results show that sequence abundance plays an important role in OTU inference. We conclude that care is needed to choose both a threshold for dissimilarity and abundance for OTU inference.

## Introduction

Microbes are estimated to have approximately 5×10^30^ cells on earth and more diverse than any other organisms [1]. They play a vital role in almost all biological processes in ecosystems from natural environments to human body [2]–[8]. Traditional culture-dependent microbial studies have limited our understanding of microbial communities, because only less than 1% of microbial organisms can be cultivated, identified, and characterized [9]. In recent years, with the development of next-generation sequencing technology, it is now possible to bypass the cultivated-based technology to sequence millions of sequences directly from relevant environments, *e.g.* human gut, soil, and salt lake. 16S rRNA sequences, the small unit of ribosomal RNA in prokaryotes, are the most widely used sequences for inferring the phylogenetic relations among microbial species [10]–[13]. The 16S rRNA based phylogenetic inference has revolutionized our view of microbial diversity and composition of many environments [14]–[17]. Many large-scale metagenomics projects have been undertaken to investigate various aspects of the microbial composition, *e.g.* Human Microbiome Project (http://commonfund.nih.gov/hmp), International Census of Marine Microbes (http://icomm.mbl.edu), and Earth Microbiome Project (http://www.earthmicrobiome.org). Thousands of 16S rRNA sequence datasets have been generated through these community efforts as well as individual projects. Therefore, there is a critical need to develop and evaluate efficient and accurate computational algorithms to analyze these massive data collected from various biological and ecological environments.

Two approaches are commonly used to characterize microbial communities in the analysis of 16S rRNA sequences: taxonomy-dependent methods and OTU-based methods [18]–[22]. The taxonomy-dependent methods rely on the annotated sequences already deposited in the databases for taxonomic assignment of a query sequence by the best-matching sequence in the reference database. In the OTU-based methods, all the sequences are clustered into OTUs based on a distance matrix at a specified threshold. Although taxonomy-dependent methods can assign taxonomy to the query sequences based on previously characterized microbes, lack of sufficient well-characterized microbes and reliable taxonomy often make it difficult to characterize novel sequences, and the robustness and accuracy of such methods are mainly dependent on the completeness of the annotated reference database [16], [20], [22], [23]. Another limitation of the taxonomy-dependent methods is that most existing reference databases are well-characterized only at the genus level or higher, rather than at the species level. In contrast, OTU-based methods are able to assign all sequences into OTUs without prior information of the reference taxonomy. Hence all sequences can be processed, including both microbes that have not been annotated in the databases as well as novel uncultured ones. The OTU-based methods are especially useful in analyzing less characterized microbial communities. Yet some issues that are unique to the OTU-based methods need to be addressed for their successful applications, such as the presence of sequencing errors which would result in an inflation of OTUs, the heterogeneous evolution rates in 16S rRNA which make it difficult to choose a consistent threshold to define OTUs, and biologically meaningful interpretations/annotations of the inferred OTUs.

In general, the OTU-based methods can be categorized into hierarchical clustering, heuristic clustering and model-based clustering methods. In the hierarchical clustering category, a distance matrix measuring the difference between each pair of sequences is calculated first, and standard hierarchical clustering is then used to define OTUs at a specific level of sequence dissimilarity. Most of these methods have an *O*(*N* ^2^) computational complexity, where *N* is the number of sequences, posing a significant computational bottleneck for processing large-scale sequencing datasets. The representative hierarchical clustering algorithm is Mothur [19], which is an improved version of Dotur [15]. Considering that the average-linkage method is relative conservative, Huse et al [24] proposed an improved single-linkage preclustering method (SLP) which can mitigate the effect of “noise” due to sequencing errors and the impact of abundant sequences so as to reduce the number of inflated OTUs. In order to reduce the complexity and memory requirement, Sun et al. [21] developed a new algorithm named ESPRIT, which adopts a k-mer distance to filter out large amount of unnecessary sequence pairs and store the reduced-distance with a sparse matrix. In addition to the filtering process, they introduced an hcluster algorithm to perform complete-linkage clustering, which reduces the computational burden so that it can process millions of sequences at one time. However, this method still has a quadratic complexity. Recently, these authors proposed an improved version ESPRIT-Tree [22], a learning-based algorithm, which may achieve similar accuracy as ESPRIT but a quasi-linear computational complexity. Overall, the hierarchical clustering approaches may not be suitable for dealing with large-scale sequencing data because of their intrinsic computational complexity. As a result, greedy heuristic algorithms have been proposed to assign sequences into OTUs, which can substantially reduce the time and space complexity compared to a quasi-linear algorithm. The most commonly used heuristic clustering methods are CD-HIT [25] and Uclust [26]. They share many features while differ on how the sequences are sorted and mapped to existing cluster representative sequences. For a pre-defined threshold, these two algorithms first select an input sequence as a seed for the initial cluster, and then examine each input sequence sequentially. If the distance between the query sequence and representative sequences of the existing clusters is within the pre-defined threshold, the input sequence will be added to the corresponding cluster, otherwise a new cluster is created and the query sequence is stored as a new seed. Based on a grammar distance metric, Russell et al. [27] proposed a sequence clustering algorithm GramCluster, which has a memory complexity of *O(LN)*, where N is the number of sequences and L is the average sequence length. Considering the computational efficiency and scalability, Ghodsi et.al [28] presented a greedy clustering algorithm named DNAClust which incorporates a novel k-mer filtering algorithm to avoid most pairwise alignments. Heuristic clustering algorithms achieve a lower complexity at the cost of reduced biological accuracy, that is, there is a trade-off between complexity and accuracy [22], [28] between the hierarchical clustering and heuristic clustering methods. In defining OTUs, some studies showed that it is difficult to use a consistent threshold because there is considerable overlap in the maximum intra-taxon distance between taxonomic levels [20]. Lastly, to avoid using a hard threshold value in clustering as implemented in hierarchical and heuristic methods, Hao et al [29] proposed a Gaussian mixture model-based clustering algorithm termed Clustering 16S rRNA for OTU Prediction (CROP). It adopts an unsupervised probabilistic Bayesian clustering algorithm and uses a soft threshold for defining OTUs. The CROP algorithm bypasses setting an often subjective hard cut-off threshold thus may effectively reduce the effects of PCR and sequencing errors in inferring OTUs.

With the availability of numerous OTU-based algorithms, it is a challenge for practitioners to choose an appropriate method for clustering their collected sequences into OTUs. Sun et al [21] studied the impact of alignment on OTU estimation. They found that pairwise alignments yielded a smaller distance than those from multiple alignments so that the use of pairwise alignment could reduce the inferred OTU number. Later, they illustrated the behavior of hierarchical and heuristic clustering algorithms in OTU construction, and concluded that hierarchical clustering algorithms are more accurate [17]. Huse et al [24] evaluated three different hierarchical clustering algorithms, and showed that the choice of clustering strategy could significantly affect the number of estimated OTUs. Overall, average-linkage clustering is more robust than complete-linkage clustering while single-linkage is rarely used because of its chaining effect [30]. Another study also showed that average-linkage clustering could provide better results and lower the effects of sequence errors in OTU estimation, yet it still overestimated the number of OTUs [31]. Schloss [32] examined some other factors such as distance calculation and sequence filtering methods which may affect the results in processing the 16S rRNA data. A more recent work by Schloss and Westcott [20] assessed the performance of four OTU-based algorithms on RDP-based benchmark datasets, and concluded that it is difficult to set a consistent threshold for defining OTU. Moreover, they introduced a novel heuristic clustering method (phylotype-OTU) with taxonomic information that can reduce the computation complexity without sacrificing the robustness of OTU assignments. This study is limited in the number of methods compared, and the choice of the RDP-based benchmark datasets that could not effectively reflect the nature of the data commonly used for OTU inference. For example, the 454 read lengths are often shorter than the 450 bps analyzed in the paper and sequencing errors were not explicitly considered. In order to investigate whether the secondary structure information would affect the estimation of OTUs, Wang et al., [33] used simulations to show that incorporating such information does not improve the OTU assignments. However, Schloss contested this point by more strict experiments [34]. Most recently, Sun et al [30] reported a large-scale benchmark study that evaluated seven OTU algorithms based on normalized mutual information (NMI) [35]. A benchmark dataset was constructed based on RDP [36] and Taxcollector [37]. Yet, it is not a strict criterion because the sequence divergence is not distributed evenly along the 16S rRNA gene and the retained sequences at a fixed 97% identity may only partially agree with the ground-truth. Meanwhile, some reads were annotated by multi-species, i.e. one-to-many mapping. However, the relative performance of different methods may depend on these processing steps to create the benchmark dataset. In addition, it is recognized that abundance sequences are more likely to be generated from true sequences, yet the rare biosphere may not be as large as previously assumed [38] because many such units may be spurious, potentially from the accumulation of small sequence errors.

Despite many published studies discussed above on the developments and evaluations of various OTU inference methods, only several papers haven discussed how to choose an appropriate method for analyzing 16S rRNA sequences and more needs to be done to provide a comprehensive assessment of different methods. This is because many of these studies did not have ground truth, assumed error-free sequencing data, and did not consider the impact of sample complexity and abundance information on OTU inference. In this article, we compare ten OTU-based methods using both simulated and real data, where ground truth is known. We introduce errors in our simulated sequence reads to mimic real data settings. Sample complexity and abundance information is also explicitly considered in our comparisons. Our results suggest that when the default parameter settings are used, the commonly used methods tend to inaccurately estimate the number of OTUs. Therefore, it is important to choose proper parameters when applying a clustering algorithm for OTU inference. In addition, we note that the abundance of reads plays an important role on OTU clustering and the appropriate abundance thresholds depend on individual clustering algorithms. Our results may provide general guidelines on selecting an appropriate method for 16S rRNA data in microbial analysis.

## Methods

### Data sets

In our study, eleven datasets (both real and simulated) were used to evaluate ten existing OTU-based methods.

### Real data (Clone43)

The real data considered was described by Huse et al [39]. This study sequenced the V6 region of 16S rRNA from a community of 43 known microbial species. It consists of 202,340 reads with read length ranging from 57 to 145 bps.

### Simulated data

Since it is difficult to evaluate the performance on a complex community because of the lack of ground-truth (e.g. the number of microbial species and their relative abundance), we used simulated datasets so that the species origin of each individual sequence read and the species abundance are known. To make the simulations more representative of nature samples, we considered simulations with different complexities, as reflected by the number of species (ranging from 10 to 200), the composition of the species (e.g. whether it is dominated by similar or distant species), and the read lengths (Table 1). To cover a wide range of scenarios, we generated ten 16S rRNA simulated datasets (simclone10_1, simclone10_2, simclone15_1, simclone15_2 and simclone20, simclone30, simclone50, simclone100, simclone150, simclone200) using the software 454Sim [40], which simulates 454 data using configurable statistical models that can accommodate different number of sequence reads, sequence lengths, errors rates, and abundance. Table 1 provides the detailed information for the simulated datasets, including the number and the length of species, the minimum and maximum distance among these species, simulated cluster size and other information. For example, Simclone10_1 and simclone10_2 were generated from 10 known species sequences but have a different distribution of reads length and relative abundance. Simclone15_1 and simclone15_2 were generated from 15 known species, simclone20 was generated from 20 known species sequences.

**Table 1:** Details on the simulated datasets.

	Num. species	Species length	Speices similarity arrange (%)	Total reads	Initial Abundance ratio (%)
Simclone10_1	10	226∼252	70.00∼93.00	69958	[6.25,3.89,5.06,11.31,7.74,29.77,8.04,12.50,5.95,9.52]
Simclone10_2	10	218∼255	70.59∼92.94	148374	[6.13,8.02,4.25,9.91,9.43,14.62,10.38,12.26,11.79,13.21]
Simclone15_1	15	59∼71	50.68∼83.04	63616	[3.57,1.78,1.78,5.35,3.57,17.86,3.57,7.14,1.79,1.79,5.36,3.57,10.71,14.29,17.86]
Simclone15_2	15	59∼82	50.68∼82.54	134092	[7.58,5.30,12.12, 18.94, 3.79, 8.71, 17.05, 3.79, 8.71,14.02]
Simclone20	20	64∼261	25.58∼94.19	115654	[2.87,1.97,2.12,4.54,4.84,9.53,6.05,6.05,3.02,2.42,3.18,5.45,3.18,7.57,8.62,6.81,5.45,4.84,8.62,2.87]
Simclone30	30	212∼241	69.72∼94.44	128308	[4.41,5.26,1.07,4.21,2.98,0.96,1.73,3.07,4.56,5.45,1.12,4.92,4.76,2.66,3.93,1.02,2.10,4.90,4.56,4.48,3.46,0.85,4.62,4.77,3.31,4.01,3.55,2.25,3.87,1.14,]
Simclone50	50	210∼242	69.86∼95.85	152373	[1.22,3.10,2.78,0.68,0.58,1.70,3.50,1.46,2.19,0.90,3.22,1.09,1.87,3.08,3.41,3.72,2.18,0.68,0.81,0.97,3.21,0.99,3.15,1.10,4.06,1.27,0.85,1.19,2.20,1.86,1.66,3.53,2.16,1.94,3.72,1.19,3.21,3.39,1.42,2.07,0.44,0.38,2.38,2.95,3.93,0.62,2.47,1.91,0.21,1.38]
Simclone100	100	212∼276	67.03∼95.93	248968	[0.14,0.94,1.55,0.72,1.44,0.89,0.62,0.91,1.29,0.97,0.37,1.76,0.70,0.43,1.02,0.48,0.32,1.12,1.25,0.81,1.04,0.36,0.94,0.42,0.57,0.60,0.32,1.44,0.45,0.89,1.15,0.81,1.02,1.47,1.38,0.70,0.65,1.01,0.46,1.62,0.41,0.40,1.48,1.20,1.68,0.86,0.72,0.80,0.38,1.46,1.80,1.02,1.25,1.77,1.29,1.09,1.54,0.80,1.74,1.62,1.06,1.70,1.28,1.56,1.13,1.50,0.38,1.58,0.57,1.01,1.13,0.68,1.27,1.76,0.99,0.75,0.37,1.39,0.34,0.77,1.25,0.44,0.94,0.48,1.48,1.23,1.24,0.97,1.58,1.17,1.41,1.58,0.51,0.91,0.58,1.47,0.42,1.16,0.91,0.68]
Simclone150	150	211∼276	67.03∼96.97	359153	[0.09,0.64,1.03,0.50,1.00,0.61,0.40,0.64,0.89,0.68,0.25,1.17,0.47,0.29,0.76,0.32,0.21,0.75,0.90,0.55,0.73,0.24,0.65,0.30,0.41,0.41,0.23,1.00,0.31,0.62,0.82,0.57,0.71,1.01,0.97,0.52,0.47,0.69,0.33,1.09,0.28,0.27,1.02,0.82,1.11,0.61,0.51,0.56,0.28,0.97,1.20,0.72,0.86,1.18,0.87,0.76,1.09,0.56,1.19,1.11,0.72,1.16,0.91,1.06,0.78,1.04,0.25,1.05,0.40,0.68,0.77,0.48,0.85,1.17,0.71,0.50,0.26,0.92,0.21,0.53,0.83,0.30,0.65,0.33,0.99,0.84,0.86,0.69,1.07,0.77,0.93,1.06,0.34,0.62,0.43,0.99,0.29,0.82,0.64,0.48,0.72,0.34,0.38,1.32,0.62,0.36,0.25,1.20,1.27,0.60,0.27,1.10,0.59,0.34,0.85,0.25,0.22,0.77,1.24,0.38,0.30,0.21,0.88,0.95,1.17,0.46,0.86,0.27,0.32,0.65,0.72,0.32,0.91,0.70,0.75,0.88,0.31,0.84,1.20,0.60,0.29,0.24,0.19,0.54,0.41,0.55,0.86,0.82,0.49,0.65]
Simclone200	200	190∼276	64.26∼96.97	484404	[0.06,0.47,0.77,0.37,0.75,0.46,0.31,0.48,0.65,0.50,0.19,0.87,0.35,0.22,0.54,0.24,0.16,0.57,0.65,0.42,0.54,0.19,0.49,0.22,0.29,0.30,0.18,0.74,0.22,0.46,0.61,0.43,0.52,0.75,0.71,0.38,0.35,0.51,0.24,0.80,0.19,0.20,0.76,0.61,0.83,0.45,0.38,0.40,0.19,0.73,0.88,0.52,0.63,0.86,0.64,0.55,0.82,0.40,0.86,0.82,0.53,0.86,0.65,0.78,0.58,0.76,0.19,0.79,0.30,0.51,0.58,0.35,0.63,0.87,0.53,0.38,0.20,0.70,0.16,0.41,0.60,0.23,0.47,0.24,0.75,0.62,0.64,0.50,0.80,0.59,0.69,0.77,0.25,0.46,0.32,0.71,0.21,0.59,0.47,0.35,0.53,0.26,0.28,0.98,0.47,0.26,0.18,0.88,0.93,0.44,0.18,0.81,0.42,0.25,0.61,0.18,0.17,0.59,0.92,0.28,0.23,0.14,0.66,0.70,0.87,0.33,0.63,0.19,0.24,0.49,0.53,0.23,0.66,0.52,0.56,0.63,0.25,0.62,0.90,0.45,0.21,0.16,0.14,0.39,0.31,0.41,0.62,0.60,0.37,0.48,0.45,0.77,0.46,0.43,0.56,0.56,0.16,0.27,0.47,0.83,0.34,0.65,0.16,0.14,0.62,0.68,0.43,0.51,0.46,0.76,0.62,0.31,0.91,0.48,0.40,0.41,0.86,0.56,0.51,0.81,0.28,0.27,0.37,0.14,0.89,0.18,0.15,0.84,0.76,0.82,0.82,0.18,0.33,0.63,0.92,0.60,0.54,0.75,0.62,0.44]

### Clustering reads into OTU

We compared ten OTU-based methods from three broad classes: hierarchical clustering (Mothur [19], ESPRIT [21], ESPRIT-Tree [22], SLP [24], Muscle [41]+Mothur), heuristic clustering (CD-HIT [25], Uclust [26], GramCluster [27], DNAClust [28]) and model-based clustering methods (CROP [29]). For hierarchical clustering and heuristic clustering algorithms, we clustered reads into OTUs at or equal to dissimilarity thresholds ranging from 1% to 10% with an increment of 0.01 regardless of the definition of dissimilarity in different algorithms. For the model-based clustering method, CROP, reads were clustered into OTUs with a soft threshold, which ranged from 0.01 to 0.10. For the Muscle+Mothur method, Muscle is multiple-alignment program rather than a clustering algorithm. Hence we used Muscle (with the default parameters) to align the reads, and then the average neighbor algorithm in Mothur was used to perform the clustering (when we calculated the distance matrix for alignments from muscle, a gap was only penalized once, and terminal gaps were penalized). For Mothur, we used the pairwise.seqs command to obtain the distance matrix which adapted the Needleman-alignment algorithm to align sequences, and then the average neighbor algorithm was used to perform clustering. To make relatively fair comparisons across different methods, we used the default parameter settings for all the OTU-based algorithms in our analysis.

### Assessment of clustering quality

In order to generate robust statistical results, inspired by the concept of Q-CV test [42], we repeated the experiment 5 times for each simulated data set. For each iteration, 90% of the reads were randomly extracted from the simulated dataset. We evaluated the method performance with different metrics. We first examined the number of estimated OTUs, and then assessed the cluster quality using precision, recall and NID [43], respectively. Precision is defined by the number of reads that are both in class *i* and cluster *j*, divided by the number of the reads in cluster *j*, thus it measures the homogeneity of cluster *j*. Recall is defined as the proportion of reads from class *i* that present in cluster *j*, thus it measures the completeness. Precision and recall provide useful insight on local performance. NID is an information theoretic-based measurement that can assess the cluster globally with a nominal [0, 1] range.

Now we define these measures more formally. Assume that there are N reads from m species 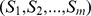
and they are clustered into n clusters 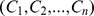 at specific dissimilarity threshold by a given clustering algorithm. Let |*S_i_*| denote the true number of reads from species *i,* |*C_j_*| denote the number of reads from cluster *j*, and *a*_ij_ denote the number of reads from species *i* and categorized into cluster *j*.

Using the above notations, precision and recall are defined as:
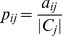


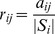



NID is defined as:
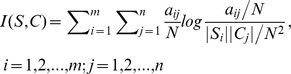


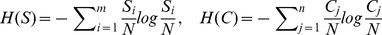


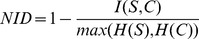



Smaller NID values imply better clustering results. It stratifies both normalization and metric properties and has a tighter bound than other measures such as NVI, NMI_joint_ [43] and thus is a useful measure for cluster validation.

## Results and Discussion

### Inferred number of OTUs

For the real dataset Clone43 and the simulated dataset Simclone15_1, Table 2 summarizes the inferred numbers of OTUs with the dissimilarity threshold set at 2%, 3% and 4%, respectively, together with the true numbers of OTUs. The results for Simclone10_1, Simclone10_2, Simclone15_2, Simclone20, Simclone30, Simclone50, Simclone100, Simclone150 and Simclone200 are shown in Table S1. Although sequence divergence is not evenly distributed in the 16S rRNA region, 3% dissimilarity is often chosen in practice as the cutoff value to define bacteria species [2], [21], [32], [39]. At this dissimilarity level, for the real dataset Clone43, ESPRIT gave the largest estimated number of OTUs (4,397), nearly 100 times larger than the expected 43 OTUs, while CROP yielded the smallest number of estimated OTU (133), still nearly three times of the true number. For the simulated dataset Simclone15_1, we found that CROP returned 15 OTUs, equal to the true number of OTUs, and SLP returned 17 OTUs. GramCluster yielded 225 clusters, the largest estimated number of OTUs among all the methods. Similar results were found for simclone15_2 which was simulated from similar species distribution but different initial abundance with simclone15_1. For simulated datasets simclone10_1, simcloen10_2, and simclone30, these methods also tended to overestimate the number of OTUs at 3% dissimilarity except the SLP algorithm, yet, the extent of overestimation is smaller when it is compared with simclone15_1 and simclone15_2. This may be due to the fact that the length of template species generated for these three simulated data sets are longer and they share a strict inter-species similarity range, a smaller upper-limit but higher lower-limit similarity, so they can tolerant more sequence errors in clustering. The results for Simclone20 were similar to those of simclone30, which have similar species distribution but with an overall lower inter-species similarity. These results suggest that the upper-limit rather than lower limit of inter-species similarity plays an important role on clustering results. However, the results are different for the other four complex simulated datasets. When we inferred OTUs at 3% dissimilarity, most hierarchical algorithms underestimated the number of OTUs. For example, CROP returned 122 OTUs, SLP returned 144 OTUs and ESPIT-Tree returned 175 OTUs for the simclone200. A more interesting finding is CD-HIT, which inferred the true (or close to the true) number of OTUs, and outperformed hierarchical clustering algorithms. This is partly due to the fact that these four data sets were generated from similar species populations. This suggests that if we choose the commonly used 3% dissimilarity to define OTUs at the species level, we may end up with an incorrectly estimated (overestimated or underestimated) number of OTUs, thus a constant threshold (e.g. 3% dissimilarity) is not ideal. Therefore, the choice of dissimilarity for defining the taxonomy is both dependent on the cluster algorithms as well as on the complexity of the dataset. For comparisons between different algorithms, we found that SLP returned a smaller number of OTUs for the simulated datasets. This could be explained by the chaining results of SLP [30], which adapts a single-linkage pre-clustering (2%), thus, more sequencing errors can be tolerated with longer reads. Mothur inferred a smaller number of OTUs than Muscle+Mothur, consistent with the argument that pairwise distances tend to yield more biologically meaningful OTUs than those with multiple alignment distances [21]. Among the hierarchical clustering methods, SLP returned the smallest numbers of OTUs mainly due to the use of precluster which could reduce the spurious clusters generated by the erroneous sequences. Unfortunately, it often led to another problem. If the data sets are generated from near-clonal populations, it is sometimes difficult to differentiate similar species due to sequence errors. In the context of inferring the number of OTUs, CD-HIT performed much better than GramCluster and DNAClust regardless of the complexity of the simulated data sets. In general, at the same cluster scale referred to the cluster thresholds, the hierarchical clustering algorithms returned smaller numbers of OTUs than heuristic clustering methods. The exception is ESPRIT, which adapts a complete-linkage (default) clustering to group reads into OTUs. It applies a more stringent threshold so that no sequence can be added to an existing OTU unless the distances between the new sequence and the sequences already in the OTU are smaller than the threshold. For CD-HIT and Uclust, consistent with what was reported by Cai et al. [22], these two methods ran several orders of magnitude faster than the hierarchical clustering algorithms at the cost of accuracy. The Gaussian model-based clustering algorithm CROP achieved the best result in the inferred number of OTUs for lower complex data sets (such as data sets simclone10_1 and simclone10_2) but had worse performance for highly complex data sets (such as simclone200), where it under-estimated the numbers of OTUs.

**Table 2:** Numbers of inferred OTUs from different dissimilarity thresholds for different algorithms.

	Clone43				Simclone15_1			
	Expected OTUs	Inferred* OTUs(2%)	inferred OTUs(3%)	inferred OTUs(4%)	Expected OTUs	inferred OTUs(2%)	inferred OTUs(3%)	inferred OTUs(4%)
Mothur	43	1882	720	369	15	63	41	20
Muscle+Mothur		2478	1418	784		117	89	54
ESPRIT		4474	4397	1733		131	131	55
ESPRIT-Tree		2301	1096	279		96	29	16
SLP		286	245	227		17	17	15
Uclust		2177	1883	597		80	75	51
CD-HIT		1473	1464	481		50	49	32
DNAClust		3768	3658	1103		239	225	53
GramCluster		2119	2071	2071		70	70	70
CROP		339	133	62		21	15	15

* : all the listed numbers of OTU are the average numbers over xx simulations.

### Relationship between the dissimilarity threshold and cluster algorithms

**Figure 1:**
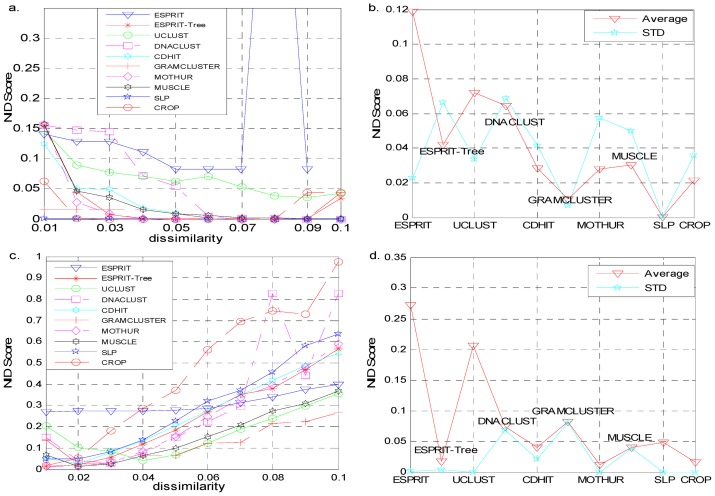
NID scores of ten algorithms based on the data set simclone15_1 and simclone_200.

From the above analysis, we found that it is difficult to use a constant threshold to define OTUs at a specific taxonomic level. To further compare the performance of different algorithms beyond the number of inferred OTUs, we calculate NID values for different dissimilarity cut-offs ranging from 0.01 to 0.1 with a step size of 0.01. Among these values, we chose the local minimum value of each algorithm and analyzed the mean and standard deviation of the NID covering the interval [0.01, Dissimilarity_minimumNID_], where Dissimilarity_minimumNID_ is the dissimilarity level where it achieved the minimum NID, to compare these algorithms (Figure 1, Figure S1). As expected, the clustering qualities of different algorithms were dependent on the clustering dissimilarity, and the minimum NID scores for different algorithms were achieved at different dissimilarity values. This may be partly due to the fact that the dissimilarity definitions used in different methods are different. Some are alignment-based while others are alignment-free. Even for the alignment-based methods, they sometimes used different strategies to treat inserts, deletes and gaps. For example, for sequences S1 = ACGGTAT and S2 = ACGGGTATAC, the similarities achieved by CD-HIT, Uclust and ESPRIT were 100%, 78% and 87.5%, respectively. It suggests that there is no consistent threshold for all the methods. For the low-complexity and short-length data set simclone15_1, CROP had the minimum NID (0) at 0.03 dissimilarity, SLP had the minimum NID (0) at 0.01 dissimilarity, Mothur had the minimum NID (0) at 0.04 and CD-HIT had the minimum NID (0.00034) at 0.07 dissimilarity. Most methods had low NID scores (< = 0.03) except Uclust (0.04) and ESPRIT (0.08). Meanwhile, SLP and CROP had a smaller average NID score with variation in the range from 0.01 to Dissimilarity_minimumNID_. Besides ESPRIT, Uclust and DNAClust, other algorithms had an average NID score smaller than 0.05. Similar results were found for the low-complexity data set simclone15_2. For the high-complexity data set simclone200, the threshold to achieve the minimum NID values skewed to the left across different algorithms and cluster qualities differed significantly for different dissimilarity thresholds. For examples, SLP, CROP and ESPRIT had the minimum NID value at 0.01 dissimilarity, whereas Muscle+Mothur and CD-HIT had the minimum NID at 0.02 dissimilarity. Similar trends were observed for simclone150, simclone100 and simclone50. These results suggest that a more stringent dissimilarity cut-off may be needed for high-complexity data sets. This is consistent with the fact when the samples consist of similar species, a higher dissimilarity threshold will incorrectly group the high-similarity species together. From the NID score, we can see that 1) Most of the current clustering algorithms could achieve a similar minimum NID value. It indicates that with a proper dissimilarity threshold for a specific algorithm, most of them have comparable results. Overall, ESPRIT, Uclust and DNAClust have poorer clustering results among these algorithms. 2) The optimal dissimilarity threshold for different methods should take the complexity of the data sets into account. For low-complexity and short-length read data, a higher dissimilarity is preferred to define OTU while for high-complexity and long-length read data, a lower threshold should be a better choice. As for the complexity of the datasets, it can be partly estimated by the distribution of the reads and the average distance among abundance reads. 3) For low-complexity and short-length read data, CROP and SLP may be preferred for their robustness to sequence errors, while for high-complexity reads, CROP should adopt smaller threshold (such as 1% for simclone200,simclone150), otherwise, it may lead to an under-estimation of number of OTUs for its over learning. 4) An interesting finding is that the NID curve for CD-HIT is similar to those of most hierarchical algorithms for high-complexity and long-length read.

### Evaluation of different methods using Precision and Recall measures

**Figure 2:**
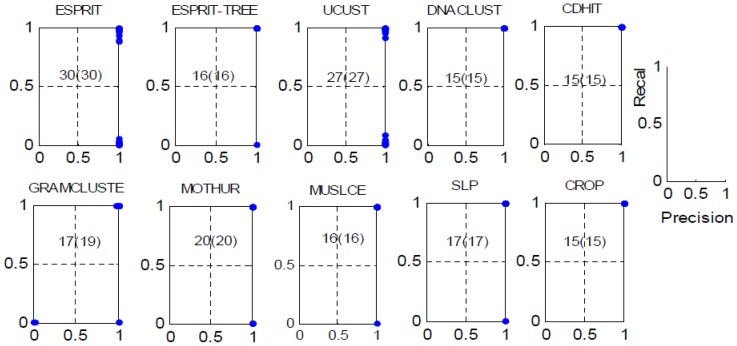
A Precision versus Recall plot generated from data set simclone15_1.

In addition to broader assessment of inferred OTU clusters in relation to the underlying community structure using the minimum NID score, we also calculated the precision and recall scores to obtain more detailed information how the sequences originating from the same species are clustered together and how the reads distribute in the clusters with different algorithms. We considered the simulated data sets where the corresponding template species for each read cloned from was known (Figure 2, Figure S2). For the dataset simclone15_1, CROP, CD-HIT, DNAClust inferred 15 OTUs with high precision and recall values which were close to 1, which suggest that these methods could cluster the reads accurately in this setting. All the other algorithms over-estimated the numbers of OTUs. The precision versus recall plots for this dataset show that some OTUs with a small cluster size and the reads are from the same species, in other words, reads from the same species were clustered into different groups. This explained the observed points appear near (0, 1). There were also a few OTUs containing reads from different species and large proportion of reads were from a dominating species while others were from different species. It can explain the points closed to (0, 0). For example, GramCluster inferred 17 OTUs and there were 19 points having non-zero (precision, recall) values, 2 out of 19 were close (0, 0). Similar results were found in other low- and moderate complexity datasets (simclone10_1, simclone10_2, simclone15_2 and simclone20). For the simulated datasets with a high complexity (such as more species and containing high-similarity species et al.), the trend became more obvious (simclone30, simclone50, simclone100, simclone150 and simclone200). 1) With an appropriate choice of threshold, the inferred number of OTUs could match the true number for all the algorithms. 2) Many OTUs generated from Uclust and GramCluster contained more than one species. ESPRIT estimated a large number of OTUs, with most clusters having a small cluster size consisting of reads from the same species. 3) Mothur, CROP, SLP, Muscle+Mothur, ESPRIT-Tree and CD-HIT achieved better performance and clustered relatively small number of OTUs, but they sometimes led to inaccurate clusters by grouping reads from different species into one cluster.

### Time cost for OTU-based methods

We noted that time cost is a critical factor when we choose an appropriate method for analyzing large scale 16S rRNA datasets. Table 3 lists the running time for each method on the dataset simclone20. Uclust took the least time to complete the clustering (0.87 minute) when the dissimilarity threshold ranged from 0.01 to 0.1, followed by ESPRIT-Tree and DNAClust (2.37 minutes and 3.01 minutes). SLP took the longest time to complete clustering (∼587 minutes). Among the heuristic clustering methods, GramCluster took the longest time to cluster sequences (36.85 minutes), due to the additional time needed to create grammar dictionary for defining dissimilarity. The running time of CROP (∼173 minutes) was shorter than that of Mothur and SLP. In general, the hierarchical clustering and model-based clustering algorithms took more time to assign sequences into OTUs than those of heuristic clustering methods.

**Table 3:** Running time for different algorithms when cluster sequences into OTUs for dissimilarity thresholds ranging from 0.01 to 0.10 based on simclone20.

	input*	Simclone20
		Running time (minute) for sequences (wall time)
Mothur	UniqueSeq	469.00
Muscle+Mothur	UniqueSeq	6.27
ESPRIT	All	75.21
ESPRIT-Tree	All	2.37
SLP	UniqueSeq	586.55
Uclust	All	0.87
CD-HIT	All	3.85
DNAClust	All	3.01
GramCluster	All	36.85
CROP	All	173.40

* : UniqueSeq represented only the unique, unaligned sequences were takes as input, All represented all sequences including the identical sequences are taken as input.

### Impact of abundance on clustering sequences into OTUs

**Figure 3:**
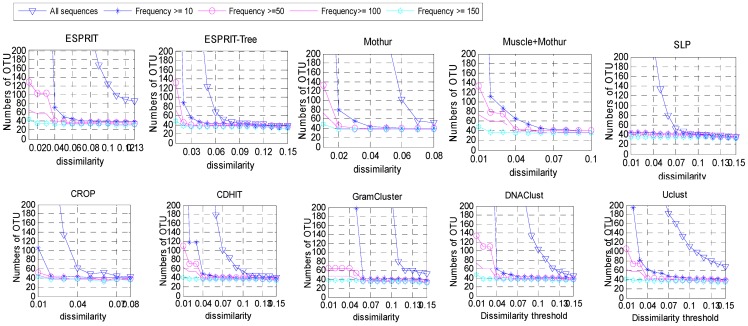
The results of OTUs estimated with different frequency thresholds at different dissimilarity levels, from the data set Clone43.

Some research showed that abundant sequences were more likely to be generated from true species while the rare sphere may be an artifact due to accumulation of sequencing errors [38]. In order to test whether abundance has an effect on OTUs estimation, we used different frequency thresholds to define abundant data set and then clustered the abundant sequences into OTUs (Figure 3, Figure S3, and Table S2). For the data set Clone43, when we used 10 as a threshold, the coverage was 93.4% and the estimated number of OTUs by ESPRIT, ESPRIT-Tree, Mothur, Muscle+Mothur, SLP, CROP, CD-HIT, GramCluster, DNAClust and Uclust was 520, 55, 56, 87, 45, 44, 118, 313, 527, and 82, respectively. With a frequency threshold 100 covering 84.2% of the total sequences, the above algorithms identified 57, 39, 40, 47, 39, 38, 53, 47, 60 and 56 OTUs, respectively, at the 0.03 dissimilarity threshold. As expected, with a higher frequency threshold, fewer OTUs were inferred. In addition, in order to recover the correct number of OTUs, different frequency thresholds may be needed according to the specific algorithms used. To illustrate these results, we analyzed five simulated datasets at different frequency thresholds and different dissimilarity thresholds to infer the numbers of OTUs (Figure S3, and Table S2). The results suggest that the abundance threshold plays an importance role in clustering reads into OTUs. For Simclone15_1 with an abundance threshold of 10, the ten algorithms inferred 30, 16, 22, 27, 15, 15, 20, 63, 20, and 21 OTUs, respectively. Similar results were obtained for Simclone15_2, with most algorithms identified the correct number of OTUs if we used 100 as the abundance threshold. Most true species were covered when we mapped OTUs to the original species. With an appropriate threshold, all algorithms can approximately recover the true number of OTUs (150 for ESPRIT, 10 for ESPRIT-Tree, SLP and CROP, and 50 for Mothur et al). Similar patterns were observed for other datasets (simclone10_1, simclone10_2 and simcloen20). It can be seen that the results from these simulated datasets were consistent with those observed with the real dataset Clone43 presented in Figure 2.

These results suggest the importance of imposing an abundance threshold in OTU inference. As for the removed reads (non-abundant reads), we may use a single-clustering or heuristic clustering method to assign the read to specific OTUs that were produced from subset of abundant reads.

## Conclusion

With rapid accumulation of 16S rRNA sequences and whole genome shotgun sequences, there is a great need to develop effective methods to analyze these data. In the past decade, taxonomy-dependent and OTU-based methods have been widely used to process the rRNA sequences. Because taxonomy-dependent algorithms depend on the completeness of existing databases while the majority of microbe species are unknown, the OTU-based methods will continue to play a vital role in microbial analysis. In this case, an accurate identification of OTU is critical for downstream analysis and biological interpretation. Although much work has been done to evaluate of the effect of the alignment, distance calculated methods, and the different variable region of 16S rRNA on OTU estimations [20], [32], relatively little guidance has been provided on the choice of a proper method for OTU estimation. In this article, we used both real and simulated datasets to compare the performance of ten existing representative OTU-based algorithms. From these results, we conclude that: 1) Most of OTU-based algorithms may either overestimate or underestimate the number of OTUs if an improper dissimilarity is chosen. The appropriate dissimilarity cut-off for inferring OTUs is not only dependent on the specific methods but also related with the complexity of the datasets. For low-complexity and short-length read datasets, a higher dissimilarity can be used to define OTU. In this case, among the ten algorithms considered, SLP and CROP achieved a better performance on OTU estimation. For high-complexity and long-length read datasets, hierarchical cluster methods need a more stringent dissimilarity threshold. Furthermore, CD-HIT has better performance similar as hierarchical clustering methods. 2) Most existing OTU-based algorithms tend to partition the samples from the same species into several sub-clusters and those OTUs sometimes have a small size, suggesting that we may reduce the overestimated number of OTUs through integrating small clusters. Unfortunately, even in the case of optimal cluster results with a minimum NID score, there also exist some OTUs with a large number of reads and coming from different species. This suggests that for these OTUs which have a large and different size from surrounding OTUs, care is needed before downstream analysis. 3) The sequence abundance plays an important role in clustering sequences into OTUs. The estimated numbers of OTUs will be reduced and become more accurate by setting a proper frequency threshold to filter out sequences with low abundance.

Existing OTU-based algorithms are sensitive to sequencing errors, it would be more effective to model sequencing errors and identify/remove “problematic” reads before clustering. This may be achieved by error calibration and modeling [44], [45]. Our results suggest the importance of choosing a proper distance threshold for taxonomic definition because of the heterogeneity of the evolutionary rate of 16S rRNA genes [20]. In addition, the dissimilarity is correlated with the distance computing methods [32]. Therefore, it is desirable to establish a taxonomic specific distance threshold that can incorporate the information on diverse evolutionary rates for 16S rRNA, and distance computing methods into consideration.

Our study has focused on the taxonomy independent clustering methods using the 454 sequence data. It is undoubtedly informative to relate each OTU to a biological entity. With the rapid accumulation of databases containing experimentally verified sequences, incorporating information of annotated sequences into OTU-based clustering methods will be critical yet few methods simultaneously analyze both annotated and unannotated sequences. Last but not least, we have focused on the analysis of 454 data and it is worthwhile to evaluate the performance with sequences generated from other sequencing platforms, e.g. Illumina, where the whole genome information is available.

## Supporting Information

Figure S1 The NID Scores of different algorithms.
(TIFF)

Figure S2 Precision versus recall plots for different datasets.
(TIFF)

Figure S3 The results of OTUs estimated with different frequency thresholds (/abundance) at different dissimilarity levels based on the dataset Clone43.
(TIFF)

Table S1 (PDF)

Table S2 (PDF)

## References

[1] Whitman WB, Coleman DC, Wiebe WJ (1998) Prokaryotes: the unseen majority. Proc Natl Acad Sci USA 95(12): 6578–6583. 9618454 10.1073/pnas.95.12.6578 PMC33863

[2] Sogin ML, Morrison HG, Huber JA, Mark WD, Huse SM, et al (2006) Microbial diversity in the deep sea and the underexplored “rare biosphere”. Proc Natl Acad Sci USA 103(32): 12115–12120. 16880384 10.1073/pnas.0605127103 PMC1524930

[3] Ley RE, Backhed F, Turnbaugh P, Lozupone CA, Knight RD, et al (2005) Obesity alters gut microbial ecology. Proc Natl Acad Sci USA 102(31): 11070–11075. 16033867 10.1073/pnas.0504978102 PMC1176910

[4] Ley RE, Turnbaugh PJ, Klein S, Gordon JI (2006) Microbial ecology: human gut microbes associated with obesity. Nature 444(7122): 1022–1023. 17183309 10.1038/4441022a

[5] Duncan KE, Gieg LM, Parisi VA, Tanner RS, Tringe SG, et al (2009) Biocorrosive thermophilic microbial communities in Alaskan North Slope oil facilities. Environ Sci Technol 43(20): 7977–7984. 19921923 10.1021/es9013932

[6] Grice EA, Kong HH, Conlan S, Deming CB, Davis J, et al (2009) Topographical and temporal diversity of the human skin microbiome. Science 324(5931): 1190–1192. 19478181 10.1126/science.1171700 PMC2805064

[7] Turnbaugh PJ, Hamady M, Yatsunenko T, Cantarel BL, Duncan A, et al (2009) A core gut microbiome in obese and lean twins. Nature 457(7228): 480–484. 19043404 10.1038/nature07540 PMC2677729

[8] Oakley BB, Fiedler TL, Marrazzo JM, Fredricks DN (2008) Diversity of human vaginal bacterial communities and associations with clinically defined bacterial vaginosis. Appl Environ Microbial 74(15): 4898–4909. 10.1128/AEM.02884-07 PMC2519371 18487399

[9] Kellenberger E (2001) Exploring the unknown: The silent revolution of microbiology. Embo Rep 2(1): 5–7. 11252724 10.1093/embo-reports/kve014 PMC1083810

[10] Lane DJ, Pace B, Olsen GJ, Stahl DA, Sogin ML, et al (1985) Rapid determination of 16S ribosomal RNA sequences for phylogenetic analyses. Proc Natl Acad Sci USA 82(20): 6955–6959. 2413450 10.1073/pnas.82.20.6955 PMC391288

[11] Pace NR (1997) A molecular view of microbial diversity and the biosphere. Science 276(5313): 734–740. 9115194 10.1126/science.276.5313.734

[12] Schloss PD, Handelsman J (2004) Status of the microbial census. Microbial Mol Biol Rev 68(4): 686–691. 10.1128/MMBR.68.4.686-691.2004 PMC539005 15590780

[13] Sharpton TJ, Riesenfeld SJ, Kembel SW, Ladau J, O'Dwyer JP, et al (2011) A High-throughput procedure quantifies microbial community diversity and resolves novel taxa from metagenomic data. PLOS computational Biology 7(1): e1001061. 21283775 10.1371/journal.pcbi.1001061 PMC3024254

[14] Eckburg PB, Bik EM, Bernstein CN, Purdom E, Dethlefsen L, et al (2005) Diversity of the human intestinal microbial flora. Science 308(5728): 1635–1638. 15831718 10.1126/science.1110591 PMC1395357

[15] Schloss PD, Handelsman J (2005) Introducing DOTUR, a computer program for defining operational taxonomic units and estimating species richness. Appl Environ Microbial 71(3): 1501–1506. 10.1128/AEM.71.3.1501-1506.2005 PMC1065144 15746353

[16] Huse SM, Dethlefsen L, Huber JA, Mark WD, Relman DA, et al (2008) Exploring microbial diversity and taxonomy using SSU rRNA hypervariable tag sequencing. PLoS Genet 4(11): e1000255. 19023400 10.1371/journal.pgen.1000255 PMC2577301

[17] Sun Y, Cai Y, Mai V, Farmerie W, Yu F, et al (2010) Advanced computational algorithms for microbial community analysis using massive 16S rRNA sequence data. Nucleic Acids Res 38(22): e205. 20929878 10.1093/nar/gkq872 PMC3001099

[18] Liu Z, DeSantis TZ, Andersen GL, Knight R (2008) Accurate taxonomy assignments from 16S rRNA sequences produced by highly parallel pyrosequencers. Nucleic Acids Res 36(18): e120. 18723574 10.1093/nar/gkn491 PMC2566877

[19] Schloss PD, Westcott SL, Ryabin T, Hall JR, Hartmann M, et al (2009) Introducing mothur: open-source, platform-independent, community-supported software for describing and comparing microbial communities. Appl Environ Microbial 75(23): 7537–7541. 10.1128/AEM.01541-09 PMC2786419 19801464

[20] Schloss PD, Westcott SL (2011) Assessing and improving methods used in operational taxonomic unit-based approaches for 16S rRNA gene sequence analysis. Appl. Environ. Microbial. 77(10): 3219–3226. 10.1128/AEM.02810-10 PMC3126452 21421784

[21] Sun Y, Cai Y, Liu L, Yu F, Farrell ML, et al (2009) ESPRIT: estimating species richness using large collections of 16S rRNA pyrosequences. Nucleic Acids Res 37(10): e76. 19417062 10.1093/nar/gkp285 PMC2691849

[22] Cai Y, Sun Y (2011) ESPRIT-Tree: hierarchical clustering analysis of millions of 16S rRNA pyrosequences in quasilinear computational time. Nucleic Acids Res 39(14): e95. 21596775 10.1093/nar/gkr349 PMC3152367

[23] Fabrice A, Didier R (2009) Exploring microbial diversity using 16S rRNA high-throughput methods. J Comput Sci Syst Biol 2: 74–92.

[24] Huse SM, Welch DM, Morrison HG, Sogin ML (2010) Ironing out the wrinkles in the rare biosphere through improved OTU clustering. Environ Microbial 12(7): 1889–1898. 10.1111/j.1462-2920.2010.02193.x PMC2909393 20236171

[25] Li W, Godzik A (2006) Cd-hit: a fast program for clustering and comparing large sets of protein or nucleotide sequences. Bioinformatics 22(13): 1658–1659. 16731699 10.1093/bioinformatics/btl158

[26] Edgar RC (2010) Search and clustering orders of magnitude faster than BLAST. Bioinformatics 26(19): 2460–2461. 20709691 10.1093/bioinformatics/btq461

[27] Russell DJ, Way SF, Benson AK, Sayood K (2010) A grammar-based distance metric enables fast and accurate clustering of large sets of 16S sequences. BMC Bioinformatics 11: 601. 21167044 10.1186/1471-2105-11-601 PMC3022630

[28] Ghodsi M, Liu B, Pop M (2011) DNACLUST: accurate and efficient clustering of phylogenetic marker genes. BMC Bioinformatics 12: 271. 21718538 10.1186/1471-2105-12-271 PMC3213679

[29] Hao X, Jiang R, Chen T (2011) Clustering 16S rRNA for OTU prediction: a method of unsupervised Bayesian clustering. Bioinformatics 27(5): 611–618. 21233169 10.1093/bioinformatics/btq725 PMC3042185

[30] Sun Y, Cai Y, Huse SM, Knight R, Farmerie WG, et al (2012) A large-scale benchmark study of existing algorithms for taxonomy-independent microbial community analysis. Briefings in Bioinformatics 13(1): 107–121. 21525143 10.1093/bib/bbr009 PMC3251834

[31] Quince C, Lanzén A, Curtis TP, Davenport RJ, Hall N, et al (2009) Accurate determination of microbial diversity from 454 pyrosequencing data. Nature methods 6(9): 639–641. 19668203 10.1038/nmeth.1361

[32] Schloss PD (2010) The effects of alignment quality, distance calculation method, sequence filtering, and region on the analysis of 16S rRNA gene-based studies. Plos computational Biology 2010 6(7): e1000844. 10.1371/journal.pcbi.1000844 PMC2900292 20628621

[33] Wang X, Cai Y, Sun Y, Knight R, Mai V (2011) Secondary structure information does not improve OTU assignment for partial 16s rRNA sequences. The ISME Journal. 10.1038/ismej.2011.187 PMC3379628 22170430

[34] Schloss PD (2013) Secondary structure improves OTU assignments of 16S rRNA gene sequences, The ISME Journal. 7: 457–460. 10.1038/ismej.2012.102 PMC3578559 23018771

[35] Studholme C, Hill DLG, Hawkes DJ (1999) An overlap invariant entropy measure of 3D medical image alignment. Pattern Recognition 32(1): 71–86.

[36] Wang Q, Garrity GM, Tiedje JM, Cole JR (2007) Naive Bayesian classifier for rapid assignment of rRNA sequences into the new bacterial taxonomy. Appl Environ Microbiology 73(16): 5261–5267. 10.1128/AEM.00062-07 PMC1950982 17586664

[37] Giongo A, Crabb DB, Davis-Richardson AG, Chauliac D, Mobberley JM, et al (2010) PANGEA: pipeline for analysis of next generation amplicons. ISME 4(7): 852–861. 10.1038/ismej.2010.16 PMC2974434 20182525

[38] Reeder J, Knight R (2009) The ‘rare biosphere’: a reality check. Nature Methods 6(9): 636–637. 19718016 10.1038/nmeth0909-636

[39] Huse SM, Huber JA, Morrison HG, Sogin ML, Welch DM (2007) Accuracy and quality of massively parallel DNA pyrosequencing. Genome Biol 8(7): R143. 17659080 10.1186/gb-2007-8-7-r143 PMC2323236

[40] Lysholm F, Andersson B, Persson B (2011) An efficient simulator of 454 data using configurable statistical models. BMC research notes 4(1): 449. 22029428 10.1186/1756-0500-4-449 PMC3214204

[41] Edgar RC (2004) MUSCLE: multiple sequence alignment with high accuracy and high throughput. Nucleic Acids Res 32(5): 1792–1797. 15034147 10.1093/nar/gkh340 PMC390337

[42] Baldi P, Brunak S, Chauvin Y, Andersen CAF (2000) Assessing the accuracy of prediction algorithms for classification: an overview. Bioinformatics 16, 412–424. 10.1093/bioinformatics/16.5.412 10871264

[43] van Rijsbergen CV (1979) Information retrieval. Boston: Butterworth.

[44] Margulies M, Egholm M, Altman WE, Attiya A, Bader JS, et al.. (2005) Genome sequencing in microfabricated high-density picolitre reactors. Nature 437, 376–380. 10.1038/nature03959 PMC1464427 16056220

[45] Richter DC, Ott F, Auch AF, Schmid R, Huson DH (2008) MetaSim – A Sequencing Simulator for Genomics and Metagenomics. PLoS ONE 3(10): e3373. 18841204 10.1371/journal.pone.0003373 PMC2556396

