# Supplementary figures and images for: A Comparison of Methods for Clustering 16S rRNA Sequences into OTUs
Source: PLoS One. 2013 Aug 13;8(8):e70837. doi: 10.1371/journal.pone.0070837 (PMC3742672; doi:10.1371/journal.pone.0070837)

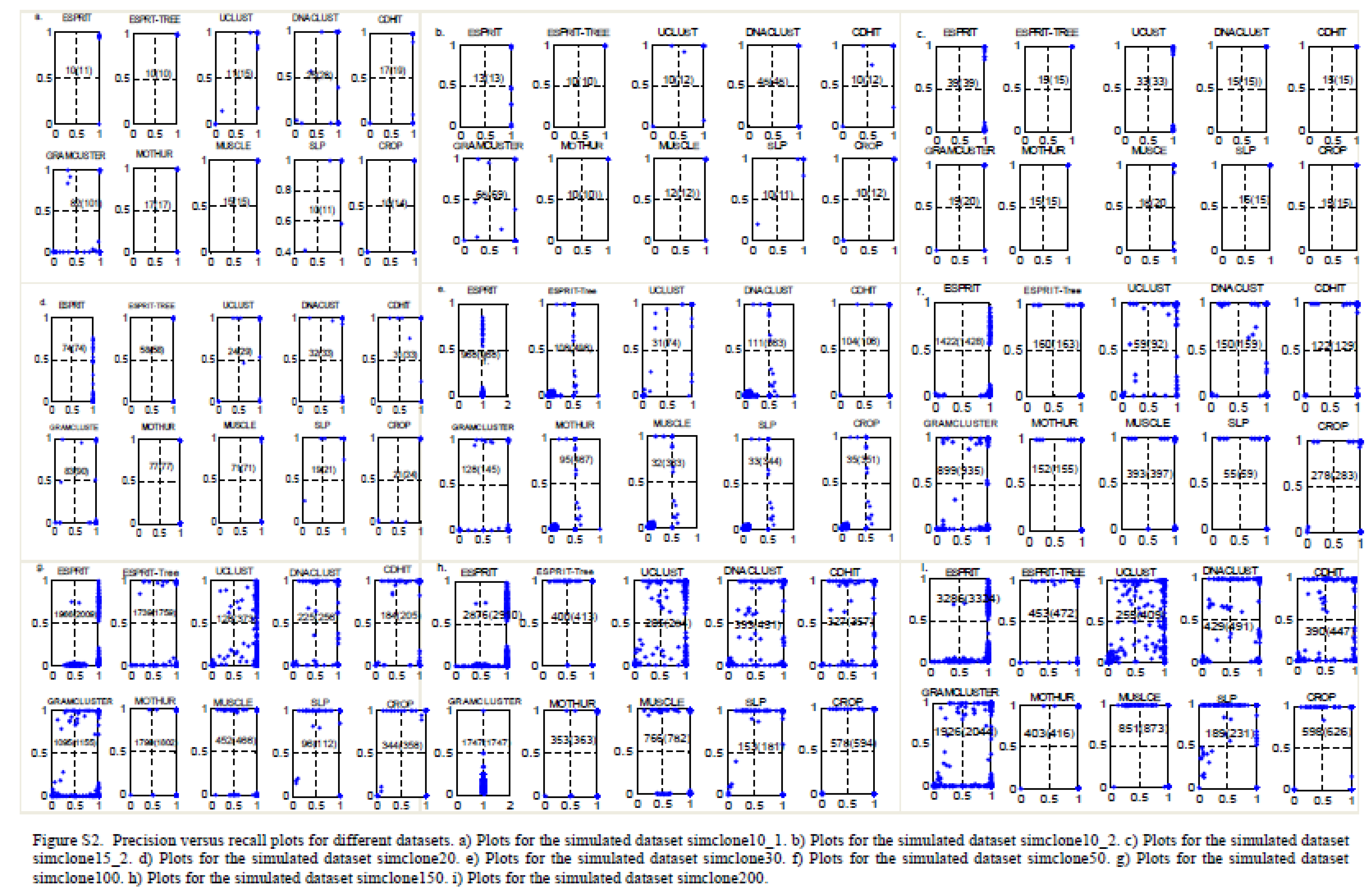

Supplement: Figure S2 — Precision versus recall plots for different datasets. (TIFF) [file pone.0070837.s002.tiff]

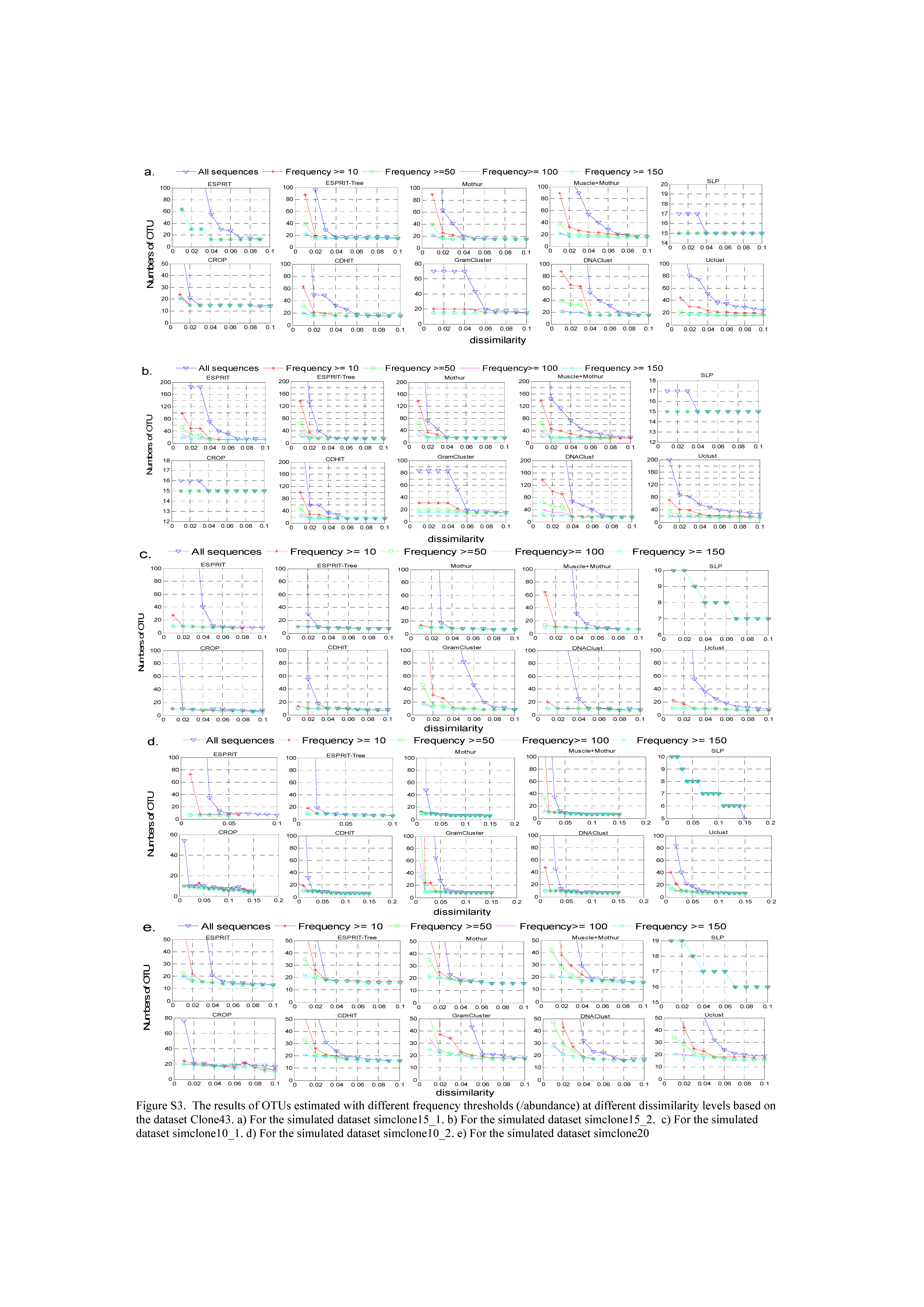

Supplement: Figure S3 — The results of OTUs estimated with different frequency thresholds (/abundance) at different dissimilarity levels based on the dataset Clone43. (TIFF) [file pone.0070837.s003.tiff]
