# Supplementary material for: A Comparison of Methods for Clustering 16S rRNA Sequences into OTUs
Source: PLoS One. 2013 Aug 13;8(8):e70837. doi: 10.1371/journal.pone.0070837 (PMC3742672; doi:10.1371/journal.pone.0070837)
Supplement: Table S1 — (PDF) [file pone.0070837.s004.pdf]

Table S1 Numbers of inferred OTUs from different dissimilarity thresholds across different algorithms

DATA SET		Algorithms									
		ESPRIT	ESPRIT-Tree	Mothur	Muscle+Mothur	SLP	CROP	CD-HIT	DNAClust	GramCluster	Uclust
Simclone15_2	Expect OTUs	15									
	inferred OTUs(2%)	184	134	73	144	16	24	59	321	84	87
	inferred OTUs(3%)	184	40	47	112	16	16	60	286	84	83
	inferred OTUs(4%)	71	20	18	73	15	16	33	68	84	57
Simclone10_1	Expect OTUs	10									
	inferred OTUs(2%)	1308	30	410	1135	10	10	55	847	973	265
	inferred OTUs(3%)	202	10	17	210	9	10	17	100	936	55
	inferred OTUs(4%)	40	8	9	31	8	8	10	25	155	36
Simclone10_2	Expect OTUs	10									
	inferred OTUs(2%)	291	19	48	190	10	10	32	228	360	84
	inferred OTUs(3%)	34	10	10	35	9	10	10	45	336	41
	inferred OTUs(4%)	13	9	9	12	8	10	10	13	65	22
Simclone20	Expect OTUs	20									
	inferred OTUs(2%)	477	58	77	269	19	21	59	396	410	133
	inferred OTUs(3%)	74	19	23	71	18	21	31	83	396	64
	inferred OTUs(4%)	21	17	19	29	17	18	24	32	83	51
Simclone30	Expect OTUs	30									
	inferred OTUs(2%)	338	108	95	240	33	35	104	440	551	161
	inferred OTUs(3%)	940	31	28	64	26	30	31	111	544	81
	inferred OTUs(4%)	31	25	26	32	23	21	29	36	128	54
Simclone50	Expect OTUs	50									
	inferred OTUs(2%)	435	160	152	393	55	57	122	654	884	237
	inferred OTUs(3%)	113	46	46	104	40	45	53	150	899	131
	inferred OTUs(4%)	44	38	36	46	35	37	45	55	218	89
Simclone100	Expect OTUs	100									
	inferred OTUs(2%)	652	235	215	452	96	100	184	845	1095	337
	inferred OTUs(3%)	212	92	93	147	75	82	99	225	1071	206
	inferred OTUs(4%)	91	64	71	92	63	70	86	106	283	128
Simclone150	Expect OTUs	150									
	inferred OTUs(2%)	1026	400	353	766	153	162	327	1424	1742	511
	inferred OTUs(3%)	338	138	145	233	112	112	149	393	1747	332
	inferred OTUs(4%)	133	96	110	132	93	71	129	171	484	205
Simclone200	Expect OTUs	200									
	inferred OTUs(2%)	1194	453	403	851	189	178	390	1668	1926	672
	inferred OTUs(3%)	395	175	185	295	144	122	196	429	1919	975
	inferred OTUs(4%)	174	128	141	185	114	84	166	202	534	255
