# Supplementary material for: A Comparison of Methods for Clustering 16S rRNA Sequences into OTUs
Source: PLoS One. 2013 Aug 13;8(8):e70837. doi: 10.1371/journal.pone.0070837 (PMC3742672; doi:10.1371/journal.pone.0070837)
Supplement: Table S2 — (PDF) [file pone.0070837.s005.pdf]

Table S2: The results of OTUs estimated with different frequency thresholds

DATA SET		# of OTU (0.03 dissimilarity)									
		ESPRIT	ESPRIT-Tree	Mothur	Muscle+Mothur	SLP	CROP	CD-HIT	DNAClust	GramCluster	Uclust
Clone43	All sequences	4397	1096	720	1418	245	133	1464	3658	2071	1883
	Abundant_10	580	55	56	87	45	44	118	527	313	82
	Abundant_50	104	40	41	75	41	40	70	110	65	73
	Abundant_100	57	38	40	58	39	38	53	60	47	56
	Abundant_150	35	36	36	37	36	36	37	38	37	38
Simclone15_1	All sequences	131	29	41	89	17	15	49	225	70	35
	Abundant_10	30	16	22	27	15	15	20	63	20	21
	Abundant_50	18	15	15	20	15	15	16	33	15	16
	Abundant_100	13	15	15	16	15	15	16	20	15	15
	Abundant_150	13	15	15	16	15	15	16	19	15	15
Simclone15_2	All sequences	184	40	47	112	16	16	60	286	84	83
	Abundant_10	49	15	27	39	15	15	27	92	31	39
	Abundant_50	27	15	15	20	15	15	17	50	20	24
	Abundant_100	19	15	15	18	15	15	16	31	16	17
	Abundant_150	14	15	15	16	15	15	16	22	16	16
Simclone10_1	All sequences	202	10	17	210	9	10	17	100	936	55
	Abundant_10	10	9	10	10	9	9	10	10	26	10
	Abundant_50	10	9	10	10	9	9	10	10	14	10
	Abundant_100	9	10	10	10	9	8	9	10	11	9
	Abundant_150	9	9	10	10	9	8	9	9	11	9
Simclone10_2	All sequences	34	10	10	35	9	10	10	45	336	41
	Abundant_10	8	9	10	10	9	10	10	10	24	11
	Abundant_50	7	9	10	10	9	9	10	10	10	10
	Abundant_100	7	9	10	10	9	8	10	10	10	10
	Abundant_150	7	9	10	10	9	9	10	10	10	10
Simclone20	All sequences	74	19	23	71	18	21	31	83	396	64
	Abundant_10	16	18	20	29	18	20	21	27	34	25
	Abundant_50	16	18	20	22	18	18	20	24	22	22
	Abundant_100	15	18	19	19	18	17	19	19	21	19
	Abundant_150	15	18	19	19	18	18	19	19	21	19

Abundant_x: the sequences in the data set with a frequency >=x
